# Typical patterns of modifiable health risk factors (MHRFs) in elderly women in Germany: results from the cross-sectional German Health Update (GEDA) study, 2009 and 2010

**DOI:** 10.1186/s12905-017-0380-4

**Published:** 2017-04-04

**Authors:** Franziska Jentsch, Jennifer Allen, Judith Fuchs, Elena von der Lippe

**Affiliations:** Department of Epidemiology and Health Monitoring, Robert Koch Institute, General-Pape-Straße 62-66, Berlin, D-12101 Germany

**Keywords:** Lifestyle, Risk factors, Health behavior, Cluster analysis, Risk clusters, German Health Update (GEDA), Elderly women

## Abstract

**Background:**

Modifiable health risk factors (MHRFs) significantly affect morbidity and mortality rates and frequently occur in specific combinations or risk clusters. Using five MHRFs (smoking, high-risk alcohol consumption, physical inactivity, low intake of fruits and vegetables, and obesity) this study investigates the extent to which risk clusters are observed in a representative sample of women aged 65 and older in Germany. Additionally, the structural composition of the clusters is systematically compared with data and findings from other countries.

**Methods:**

A pooled data set of Germany’s representative cross-sectional surveys GEDA09 and GEDA10 was used. The cohort comprised 4,617 women aged 65 and older. Specific risk clusters based on five MHRFs are identified, using hierarchical cluster analysis. The MHRFs were defined as current smoking (daily or occasionally), risk alcohol consumption (according to the Alcohol Use Disorders Identification Test, a sum score of 4 or more points), physical inactivity (less active than 5 days per week for at least 30 min and lack of sports-related activity in the last three months), low intake of fruits and vegetables (less than one serving of fruits and one of vegetables per day), and obesity (a body mass index equal to or greater than 30). A total of 4,292 cases with full information on these factors are included in the cluster analysis. Extended analyses were also performed to include the number of chronic diseases by age and socioeconomic status of group members.

**Results:**

A total of seven risk clusters were identified. In a comparison with data from international studies, the seven risk clusters were found to be stable with a high degree of structural equivalency.

**Conclusion:**

Evidence of the stability of risk clusters across various study populations provides a useful starting point for long-term targeted health interventions. The structural clusters provide information through which various MHRFs can be evaluated simultaneously.

## Background

The aging of populations around the world is a global phenomenon, receiving intensive examination in the context of social and demographic change. It is assumed that the factors and processes contributing to population aging will continue in the foreseeable future and will in fact accelerate over the next two decades [1]. Within the next 35 years, it is expected that the proportion of older people in the population will increase to about one-third [2]. While emerging data suggest a narrowing of the gender age gap in some countries [3], the longer life expectancy of women in most parts of the world [4, 5] highlights changing sex ratios associated with the process of aging. This imbalance increases with age and has been called the “feminization of age” [6, 7]. At the same time, younger birth cohorts among the elderly show steady improvement in health. Older people today live longer, on average [8], and the additional years of life are characterized by better health [5, 9, 10]. In addition to age, sex has a substantive impact on the existence of health problems. Women have a higher prevalence of chronic diseases and disorders (e.g. osteoporosis, back pain, depression) and are affected more frequently by disability and impairment [11, 12].

Modifiable health risk factors (MHRFs) such as smoking, high-risk alcohol consumption, physical inactivity, low intake of fruits and vegetables, and obesity play a substantial role in mortality rates and disease incidence among the elderly [11, 13]. According to the World Health Organization (WHO), these MHRFs are responsible for one-third of the global chronic disease burden [14] and rank among the most common risk factors leading to death in developed countries [15]. Several studies show that a “healthy” combination of MHRFs is associated with both lower morbidity rates [16–18] and lower mortality risk [19–22], and can result in substantial survival advantage.

There is evidence that MHRFs occur not only in isolation but also in specific combinations or clusters [23–34]. Different methods have been used to assess the factors associated with these risk combinations [35]. One of these methods is the application of cluster analysis. All seven of the studies examined between 1994 and 2013 that identified specific health lifestyle patterns by means of cluster analysis focused on four MHRFs: smoking, alcohol consumption, physical activity, and diet [24–26, 28, 30–32]. Only Spegel et al. [30] additionally considered body weight in their Bern-Munich Lifestyle Panel, and only one study focused on older people [28]. The results of this latter study were limited to a regional sub-sample from Baden-Württemberg, Germany and to an age range between 50 and 70 years. Obesity, however, is a serious risk factor for chronic diseases [36–38] and increased mortality [39].

A deeper understanding of the distribution of MHRFs and their combinations, and possible stabilizing relationships, should help to improve the health of older women through targeted programs of health intervention and disease prevention. This article examines typical combinations of established MHRFs in a representative sample of older women in Germany. This group in particular, because of the feminization of old age and the increased scale of medical expenses [7], forms a relevant public health population in need of study.

## Methods

### Study population

The analyses presented are based on a pooled dataset of two consecutive survey rounds (GEDA09 and GEDA10) of the German Health Update (GEDA). The GEDA study is a nationwide cross-sectional telephone survey including more than 20,000 respondents per survey round, conducted by the Robert Koch Institute (RKI) on behalf of the German Federal Ministry of Health (BMG) [40–42]. Each GEDA round was approved by The Federal Commissioner for Data Protection and Freedom of Information (BfDI), and verbal informed consent was obtained from all of the participants in advance. Information on health, health-related behaviors, living conditions, health-related quality of life, and socio-demographic factors was gathered. The results from GEDA are representative for community-dwelling adults in Germany who are reachable via landline [40–42]. The pooled dataset comprises a sample of 43,312 respondents aged 18 and older. The large sample allows analysis of subpopulations with further stratification by socio-demographic and health-related factors. The available weighting factors enable obtaining representative results according to sex, age, education, and region. The present study includes all female respondents aged 65 and older (*n* = 4,617). After excluding cases with missing information in the relevant variables, the total number of respondents left in the sample for cluster analysis was 4,292.

### Study variables

Similar to other studies that have explored patterns of MHRFs using cluster analysis [28, 30–32], all factors in this study are dichotomized so that “1” represents the presence of the risk factor (unhealthy expression) and “0” (healthy expression) its absence (see Table 1).

**Table 1:** Definitions of the five modifiable health risk factors included in the study

Risk factors	Definition of unhealthy behavior (Code = 1)
Smoking	Current smokers (includes occasional smokers)
Alcohol	High-risk alcohol consumption according to AUDIT-C
Inactivity	Less than 5 days/week with minimum 30 min/day of physical activity *and* no sports-related exercise within past 3 months
Diet	Less than 1 serving of fruits or vegetables/day
Obesity	BMI ≥30 kg/m^2^

#### Smoking

Smoking behavior was assessed by the question: “Do you currently smoke, even if it’s only occasionally?” There are four possible responses: “Yes, daily,” “Yes, occasionally,” “No, not anymore,” and “Have never smoked,” from which, in line with other studies [23, 30, 32, 33], a dichotomous “**S**moking” variable was formed. Because the group of smokers in this age group is generally very small (10%) we decided to combine the daily and occasional smokers in one category. This way the important MHRFs of smoking can be taken into account. The information on smoking includes all tobacco products.

#### High-risk alcohol consumption

To determine high-risk alcohol consumption in different cultural settings, the Alcohol Use Disorders Identification Test (AUDIT) was developed for the World Health Organization (WHO) [43] and was used in its short form, AUDIT-C, in GEDA09 and GEDA10 [41, 42]. The short form consists of three questions on alcohol consumption covering (1) how often the respondent drinks alcohol, (2) how many drinks are consumed on a typical occasion, and (3) how often the respondent drinks six or more alcohol units on one occasion. For interpretation, a sum score is formed with a maximum value of 12. For women, an AUDIT-C value of 4 or more is defined as high-risk consumption [41–43]. Although an AUDIT-C score of 3 or more is often used as an indicator of hazardous drinking, several studies recommend lowering the threshold for general population samples to provide a more sensitive and specific screen [44]. Furthermore, Dawson et al. [45] and Towers et al. [46] point out that hazardous drinking prevalence in older adult populations can be over-estimated using the standard threshold and suggest an older-adult-specific cut point of 4 [45, 46]. This definition is applied to derive the variable “**A**lcohol” in the study.

#### Physical inactivity

Following Caspersen et al., physical activity behavior was divided into physical activity and sports [47]. These two aspects were determined in GEDA09 and GEDA10 by three questions [41, 42]. The variable “**I**nactivity” was formulated according to Caspersen et al. [47] as a combination of lack of physical activity defined as recommended by the Centers for Disease Control and Prevention and the American College of Sports Medicine (to be physically active less than 5 days per week for at least 30 min) [48] and a lack of sports-related activity in the past 3 months. These cut-off points are in line with the WHO recommendations which suggest for adults aged 65 and older to be physical active (including leisure time activities as well as sports) between 150 and 300 min per week. Sports-related activity being a subset of physical activity is defined as planned, structured, and repetitive and following the goal to achieve physical fitness. Physical activity however is defined as any bodily movement produced by skeletal muscles [47].

#### Low intake of fruits and vegetables

Van Duyn et al. [49] provided scientific evidence that the consumption of fruits and vegetables has a preventive effect on the development of chronic diseases; therefore, the variable “**D**iet” as a MHRF was defined as eating less than one portion of fruit and one of vegetables per day, in general. This dichotomous variable was formed from two separate questions on consumption of fruits and vegetables [41, 42]. A portion is quantified as one handful of plant food, so that the amount consumed is adjusted to the respective constitution of the consumer [50].

#### Obesity

In accordance with the WHO classification, the variable “**O**besity” was defined as having a body mass index (BMI) greater than or equal to 30 (kg/m^2^) [51]. BMI was calculated from self-reported responses, namely, height: “How tall are you without shoes, in centimeters?” and weight: “How much do you weigh without clothes, in kilograms?”

#### Additional variables

Additional variables were included in the descriptive analysis: (1) age stratified by group (65–69, 70–74, 75–79, ≥80 years) and socioeconomic status (SES) in the form of a multidimensional SES index, including information on education, income and employment (SES graded as a continuous variable ranging from 3 to 21) [52]; and (2) similarly to Fuchs et al. [53], the number of chronic diseases and restrictions summed to a continuous variable of multimorbidity. Multimorbidity was determined as the presence of two or more concurrent health conditions in one person. Unlike Fuchs et al., however, obesity was not taken into account.

### Statistical analysis

All analyses were performed with the statistical software SPSS, version 20 (SPSS Inc., Chicago, IL, USA). To identify distinct homogeneous groups [54] among the five MHRFs in the study population, agglomerative hierarchical cluster analysis for binary data according to the Ward method was applied [55]. To develop a deeper understanding that goes beyond the structural composition of the MHRFs, extended analyses were performed taking into account the number of chronic diseases according to age and the SES.

## Results

### Distribution of modifiable health risk factors (MHRFs) and their combinations

**Fig. 1:**
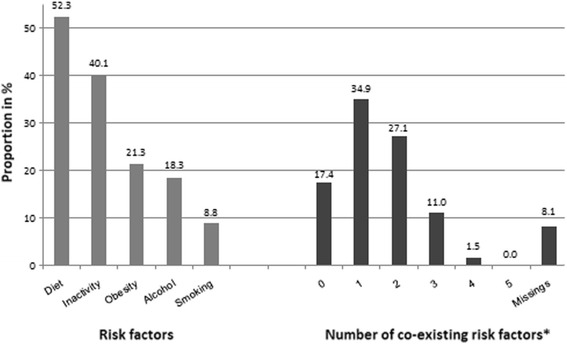
Distribution of risk factors in the study population and number of co-existing risk factors. *Calculation was made only if full information for all risk factors was given, otherwise it is considered as missing

The most prevalent MHRF among the 4,617 women aged 65 and older was “Diet” (52.3%), followed by “Inactivity” (40.1%); 21.3% were obese, 18.3% consumed alcohol in hazardous quantities, and 8.8% were current smokers (Fig. 1). Most of the older women showed at least one (34.9%) or two (27.1%) simultaneous MHRFs. The percentage of persons without any of the MHRFs was 17.4%. The simultaneous presence of four and five MHRFs among older women occurred in 1.5% and 0%, respectively (see Fig. 1). Overall, the missing information among the variables was 8.1%.

### Description of identified risk clusters

**Fig. 2:**
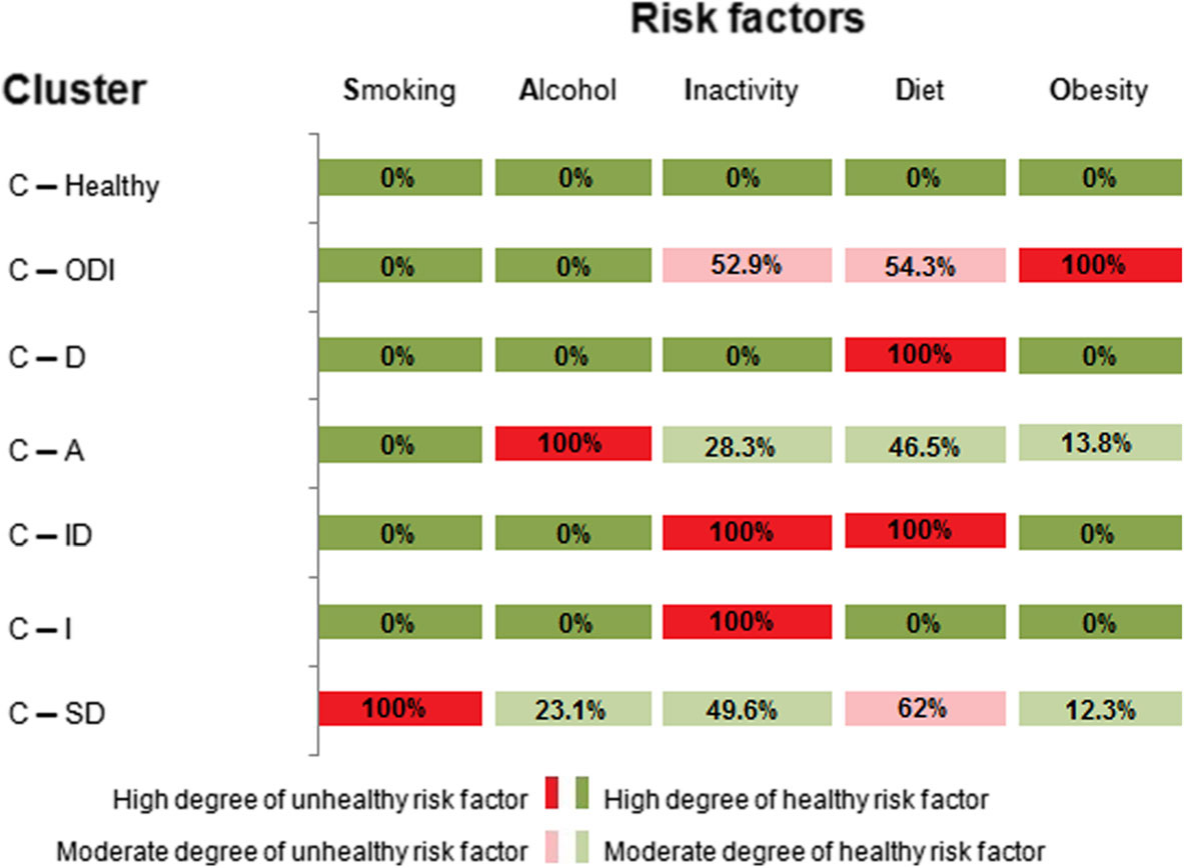
Characteristics of the seven clusters according to specific risk factors: smoking, high-risk alcohol consumption, physical inactivity, low fruit and vegetable consumption, and obesity

Using cluster analysis on the cleaned sample (*n* = 4,292), seven clusters (C) of MHRFs were identified. To obtain a quick and clear impression of the cluster compositions we developed a traffic-light system, with red symbolizing unhealthy and green healthy characteristics of the relevant MHRFs. Fig. 2 illustrates the structural composition and displays percentages for the seven clusters identified, based on the five considered MHRFs. The names of the clusters are given according to the prevalence of the MHRFs within the given clusters.

The largest cluster, with 18.9% of the study population, was “C-Healthy,” characterized by the absence of any MHRFs. “C-ODI” was the second largest cluster, with 17.4%. Women in this cluster were obese, mostly consumed very little fruits and vegetables, and were physically inactive. “C-D” was the third largest, with 17.1%, defined by only one MHRF: low consumption of fruits and vegetables. 16.5% of women were assigned to “C-A.” This cluster consisted of women with high-risk alcohol consumption, and also showed partial risk behavior in terms of physical inactivity and an unhealthy diet. Whereas some of these women were also obese, all were non-smokers. In addition to the cluster of low consumption of fruits and vegetables “C-D,” “C-ID” was also identified, consisting of women who on the one hand had low physical activity, and on the other ate too little fruits and vegetables and had no other MHRFs. This group included 12.2% of the study population.

“C-I” was, with 9%, one of the smaller clusters. Members showed a deficit only in terms of adequate physical activity. Similar in size was “C-SD,” with 8.9%. Next to smoking, the majority of this group also had low consumption of fruits and vegetables (62%) and low physical activity (49.6%). A smaller number also had risk-related drinking behavior (23.1%) and obesity (12.3%). This cluster combined all risk factors.

**Fig. 3:**
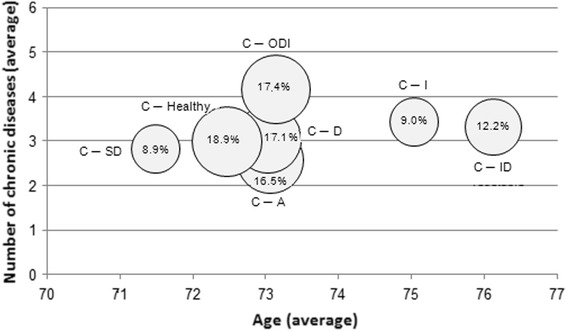
Cluster characteristics according to number of chronic diseases and age

**Fig. 4:**
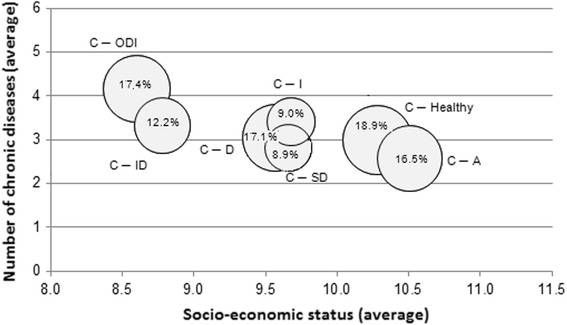
Cluster characteristics according to number of chronic conditions and socioeconomic status

The extended analyses with the number of chronic diseases according to age and the SES are plotted in Figs. 3 and 4. In both of these figures, the circles reflect the cluster size.

Fig. 3 shows that in comparison with all other clusters, the two clusters involving low physical activity (C-I and C-ID) had the highest age range, while the “smokers” cluster C-SD ranked in a “younger” age range. In terms of age, the cluster C-ODI was located in the middle of all clusters, but included the highest average number of chronic diseases. On average, women of cluster C-A had the fewest chronic diseases.

Figure 4 describes the position of the clusters in terms of the average number of chronic diseases and the average SES. After C-ID the cluster C-ODI showed the second lowest average SES, whereas the clusters C-Healthy and C-A were more likely to be related to higher SES.

**Fig. 5:**
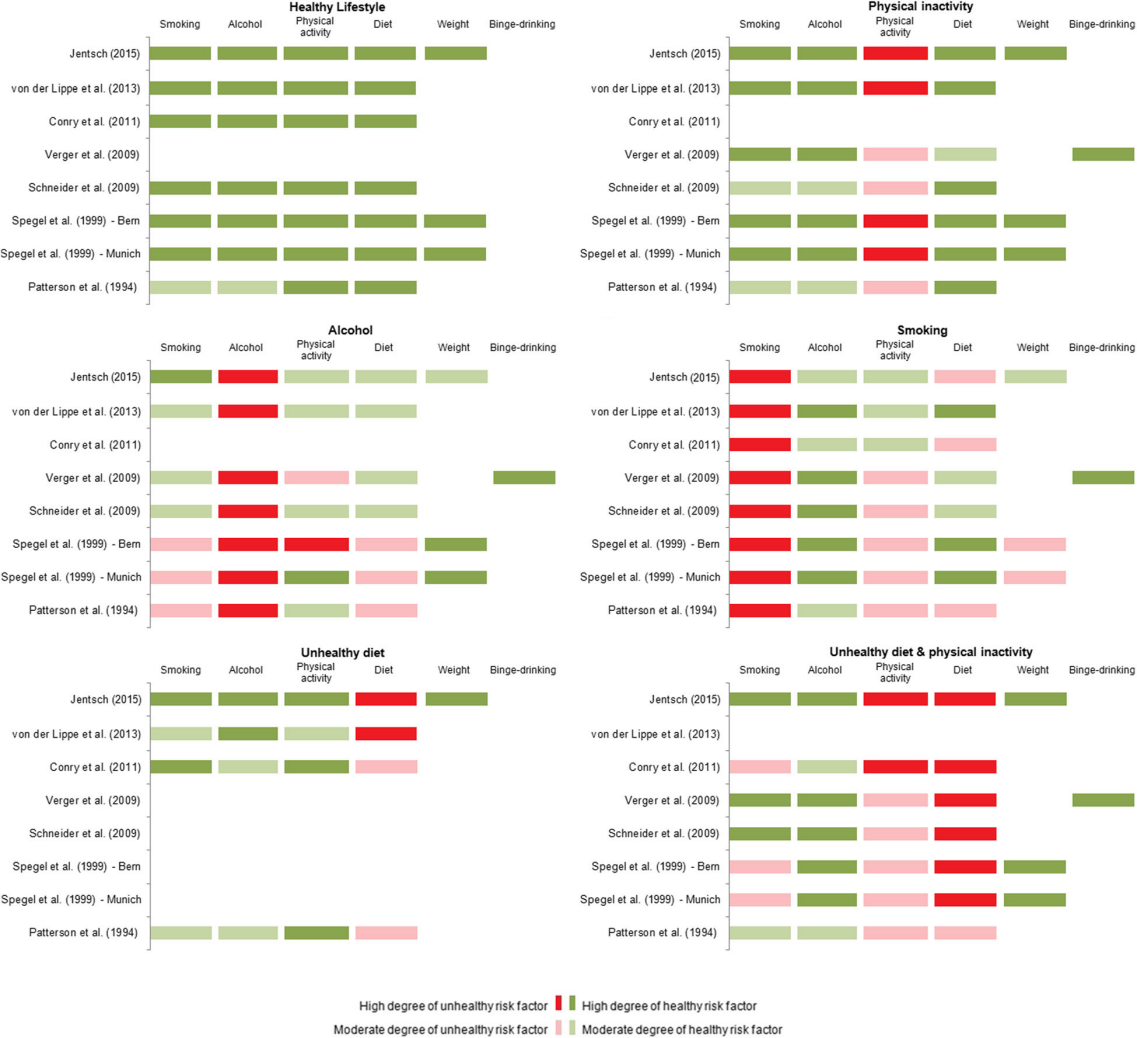
Overview of cluster solutions and their structural composition

Further, we looked at the results from other studies to classify the identified cluster solutions. The comparisons are done with studies that have comparable methodology and examine comparable MHRFs. The sample from these studies come from Germany for population 18 + [32], a region in Germany for a population aged 50–70 [28], a study from Ireland for population 18+ [24], a study from France for population 18+ [31], cohorts studies from the towns of Bern and Munich for population aged between 55 and 65 [30], and a US study for adults aged 21 and over [56]. For better visualization we present the outcome of the comparison in one figure (Fig. 5), which displays an overview of all overlapping identified cluster solutions of the studies considered and their structural composition. It should be noted that all of the studies differ in the approach to the MHRFs considered. For example, in line with others [28, 31, 32], we used low intake of fruits and vegetables instead of diet indices as one possibility to operationalize poor diet [24, 30, 56]. When the MHRF is in fact present, there are, nevertheless, differences in comparison with studies that were also limited only to consumption of fruits and vegetables.

Only Spegel et al. [30] included obesity as an additional MHRF. In contrast to the cluster C-ODI presented here, they identified two obesity clusters per study population (Munich and Bern). Because these obesity clusters differ structurally, comparable cluster solutions with obesity are missing, thus the obesity cluster is not reflected in Fig. 5.

## Discussion

With the identification of seven distinct clusters of risk patterns based on the five MHRFs: **S**moking, **A**lcohol, **I**nactivity, **D**iet, and **O**besity in older women in Germany, our study shows that MHRFs tend to be associated in clusters. Six of the identified clusters show a high structural similarity to comparable studies [24, 28, 30–32, 56], inline with a recent review [35]. The clusters C-Healthy, C-A, C-I, C-SD, C-D, and C-ID have been identified in almost all studies considered here. The denomination of the cluster solutions presented by the respective authors however differs substantially from those presented in this article.

The study closest structurally to our present work is that of Schneider et al. [28], which identified a five-cluster solution, four of which match almost exactly the structural composition of our clusters (C-Healthy, C-I, C-A, and C-ID). Only within the “Smoking” cluster did Schneider et al. find a higher proportion of physical inactivity and a lower proportion of poor diet. They were unable to identify an “Unhealthy diet” cluster [28].

The above result in particular, but also the results of the other studies, demonstrates that health risk clusters based on **S**moking, **A**lcohol, **I**nactivity, and **D**iet represent fairly stable risk patterns; that is, they are both time-stable over a period of almost 20 years and they show generally the same structure in western Europe [24, 28, 30–32] and the United States [35, 56]. This finding serves as a useful starting point for long-term intervention programs. Health promotion programs typically aim to increase the number of years of life, but campaigns targeting older people, who typically live with multiple chronic conditions, need to emphasize quality of life as well as length of life [57]. Health programs should focus on the specific health conditions observed and the specific combinations of co-existing MHRFs present. Ideally, health intervention programs don’t just target one aspect of a healthy lifestyle (for example: balanced diet), they also target the interaction between that aspect and another aspect (for example: losing weight or raising activity level).

Overall, 17.4% of the study population reported no MHRFs. In other words, in approximately four of five cases there is at least one MHRF that is not compliant with existing recommendations. Studies from the United States on adherence to health recommendations have demonstrated that education [58, 59] and higher income [58] have a positive influence on MHRFs, findings that can be extrapolated to our own detailed description of clusters regarding the average number of chronic diseases and SES (see Fig. 4). Women from the higher social classes are most likely to be in the cluster C-Healthy, a fact also reported in other studies [24, 28, 30, 32, 56], and is also true for cluster C-A. What most of the studies considered here have in common is that persons with higher SES are found in the “Alcohol” cluster [28, 31, 32, 56]. By comparison, Spegel et al. [30] found two “Obesity” clusters, with low occupational prestige as a relevant predictor of cluster membership. Membership in the C-ODI cluster in the present study is due to a lower SES that includes professional position as one of three status dimensions. In accordance with three of the five studies that also identified the cluster C-ID, individuals of that cluster were far less likely to have a high SES [24, 30, 56]. These results show that it is necessary to consider the social inequalities of people when implementing prevention and health programs. It should be recognized that people with different social statuses generally have access to different resources, have different coping strategies, and vary in terms of health literacy [60].

Comparing the detailed descriptions of clusters with the results of similar studies showed a definite overlap. In line with the literature, the members of C-I cluster are primarily older people [32, 56]; the same was found for cluster C-ID. This result has not been reported in other studies thus far. The result showing that members of the “Smokers” cluster are younger is found often in the literature [24, 28, 31].

The cluster “Obesity and all other risk factors” identified in the study population of Spegel et al. [30] showed that chronic diseases were positively associated with cluster membership. In our study population of older women, the probability of cluster membership also increased with the increasing number of chronic diseases, whereas it decreased for the alcohol cluster C-A (see Figs. 3 and 4).

### Strengths and limitations

One major strength of this study is the large and representative sample, based on the pooled dataset of GEDA09 and GEDA10. This allowed meaningful and reliable results for the subgroup of 65- to 100-year-old women. It is the first work of its kind that focuses solely on women in the older age group without an upper age limit.

We were not always able to clearly delineate the current recommendations regarding the definition of risky behavior. For example, the data collected in GEDA09 and GEDA10 did not include all the information necessary to define MHRFs following the current recommendations (e.g. physical inactivity). In addition, cut-off points for the MHRFs “Diet” and “Inactivity” were modified to increase the selectivity. To define high-risk alcohol consumption and obesity, only the scientific, unequivocally demonstrated risk characteristics were selected, owing to controversies regarding risk patterns. Accordingly, “never drinkers” and “overweight” were not included in the definition of risky behavior. Despite variation in the use of different health behavior measures and cut-off points between all six comparison studies, there is broad agreement within the cluster solutions. Furthermore, in the group of the smokers the occasional smokers are included which encompasses a wide range of smoking intensity.

A further limitation is the use of cross-sectional data, which meant that no causal conclusions could be drawn. Moreover, all data are based on self-reports, which are subject to systematic errors such as social desirability or recall bias. Presumably, the healthy cluster could be in reality smaller in size than found in the current study.

Finally, the results presented are valid for the specific female population aged 65 years and older, living in private households in Germany, and accessible via landline.

## Conclusions

Despite the varying methods used to operationalize MHRFs and the varying age ranges that appear in the studies used in for comparison, six clusters of large structural consensus can be identified in the study populations. These clusters demonstrate that MHRFs occur both separately and in specific combinations. Such risk patterns represent a useful starting point for long-term targeted health interventions. Strategies with the aim of improving specific behaviors and MHRFs can be designed based on the structural composition of each cluster, either targeting a combination of factors (e.g., the cluster C-ID) or concentrating on individual factors (e.g., C-I).

Armed with knowledge about individual MHRFs in older women, the main focus of disease prevention should be increased physical activity and encouraging a change in diet toward increased intake of fruits and vegetables to reduce the prevalence of MHRFs and risk combinations. Furthermore, weight reduction should be an expected consequence of such positive changes. Although women from the “Alcohol” cluster seem to be the “healthiest” group with the lowest average number of chronic conditions or restrictions, it is unlikely that their good health status is a result of their risky drinking behavior. Rather, women whose health has suffered only limited damage from excessive alcohol intake may be endowed with a better genetic constitution. Given the harmful effects of high-risk alcohol consumption and its dissemination in the population of older women (16.5% of women are part of the “Alcohol” cluster), this group should not be excluded when developing prevention programs. Because of the smaller size of the “Smoking” cluster and the lower prevalence of smoking in the study population, there is no identified need to develop a program related to smoking cessation and its coordinate risk factors.

Regarding trends in demographic change and the growing population of older women in Germany, the health and health behavior of these women has increasing relevance for intervention programs and public health in general. Further research is needed to better understand the interrelated factors of health and health behavior in older populations.

In conclusion, our results suggest that in the development of health intervention programs, priority should be given to three MHRFs: physical inactivity, obesity, and low consumption of fruits and vegetables, along with all their possible combinations.

## Abbreviations

A: Alcohol
AUDIT: Alcohol Use Disorders Identification Test
AUDIT-C: Alcohol Use Disorder Identification Test Consumption
BfDI: Federal Commissioner for Data Protection and Freedom of Information
BMG: German Federal Ministry of Health
BMI: Body Mass Index
C: Cluster
D: Diet
GEDA: German Health Update
I: Inactivity
MHRF: Modifiable Health Risk Factor
O: Obesity
RKI: Robert Koch Institute
S: Smoking
SES: Socioeconomic status
WHO: World Health Organization
